# Gender Differences in the abuse of new technologies, and other addiction problems of patients from primary care

**DOI:** 10.1192/j.eurpsy.2023.721

**Published:** 2023-07-19

**Authors:** F. Méndez-López, M. Dominguez-García, B. Oliván-Blázquez, C. Bartolomé-Moreno, A. Aguilar-Latorre, R. Magallón-Botaya

**Affiliations:** ^1^ Aragonese Primary Care Research Group, Aragón Health Research Institute; ^2^ Aragonese Health Service; ^3^ University of Zaragoza; ^4^Aragón Health Research Institute, Zaragoza, Spain

## Abstract

**Introduction:**

The use/abuse of Information and Communication Technologies (ICT) has become a topic of great interest in recent years. With advances in technology, today’s population spends a great deal of screen time (ST) making watching television (TV), using computers, smartphones, or playing video games a central component of their daily lives. These studies have analyzed the psychological impact of technological exposure or abuse, such as aggressive behaviors, anxiety, depression and other mental problems.

**Objectives:**

The main objective of this study is to explore the differences between men and women and the abusive use of social networks, technologies, pathological gambling and other addiction problems in primary care.

**Methods:**

This study is an observational study conducted within the framework of primary care in the Spanish region of Aragon. The population of the study were participants of 35-74 years old, had been receiving care from the Aragon Health Service. Recruitment is shown at figure 1. Sociodemographic, quality of life, personal factors on health behaviour, social support, lifestyle patterns and chronic comorbid pathology variables were collected during the period 2021–2023. The project was approved by the Clinical Research Ethics Committee of Aragon Nº PI20/302. The comparisons by sex were carried out using a Student T-test or chi squared test to analyse differences.

**Results:**

There are significant differences in the abuse of new technologies between men and women. 25.20% of men (CI 95% 18.26-33.25) compared to 13.41% of women (CI 95% 8.85-19.25) make abusive use of the Internet, with statistically significant differences. In the same way, men present greater abuse of video games (6.25% of men (CI 95% 3.0-11.45) compared to 3.05% of women (CI 95% 1.17-6.55).

Analysing the differences by sex in dependence if it is an urban or rural population. Significant differences in the abuse of new technologies between men and women are present in the urban population, while in the rural population these differences are not observed

**Image:**

**Figure:**
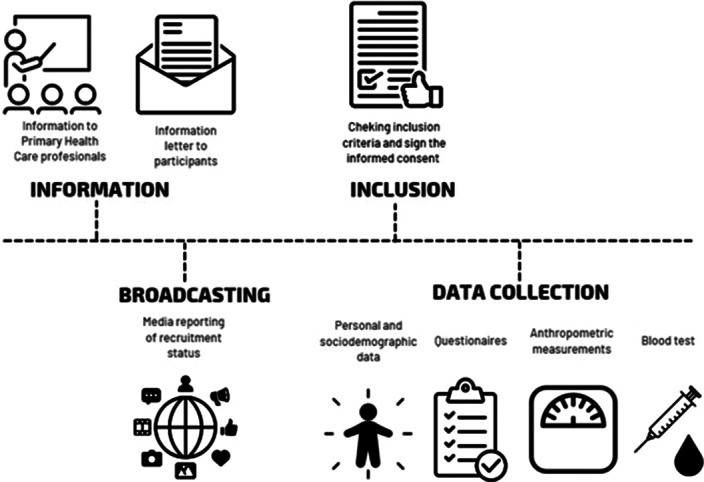

**Conclusions:**

Gender modifies the ways in which technologies are used, so that men have a more problematic use of video games and the Internet than women. On the other hand, in relation to emotional symptoms, it was observed that women presented more anxiety and less satisfaction with life than men. The evaluation of abuse of new technologies cts should be incorporated into health services to improve people’s ability their self-care, the level of knowledge of managing their disease and their physical, mental and social health.

**Disclosure of Interest:**

None Declared

